# Explainable artificial intelligence in accounting and financial auditing: a systematic review

**DOI:** 10.3389/frai.2026.1823263

**Published:** 2026-08-14

**Authors:** Iván Patricio Arias-González, Gabriela Joseth Serrano-Torres, Eduardo Ramiro Dávalos-Mayorga, Norma Patricia Jiménez-Vargas

**Affiliations:** 1 Facultad de Ciencias Políticas y Administrativas, Universidad Nacional de Chimborazo, Riobamba, Ecuador; 2 Facultad de Ingeniería, Universidad Nacional de Chimborazo, Riobamba, Ecuador

**Keywords:** fraud detection, LIME, machine learning, SHAP, XAI

## Abstract

Explainable Artificial Intelligence (XAI) has emerged as a response to the need to understand and make transparent the decisions of machine learning models, particularly in sensitive contexts such as accounting and financial auditing. In this domain, XAI enables the interpretation of results generated by automated systems applied to fraud detection, risk management, financial analysis, and regulatory compliance, thereby strengthening the trust of auditors and regulators. The objective of this study was to systematically analyze the literature on XAI in accounting and financial auditing in order to identify its application domains, the methods employed, and the main challenges reported. The research was conducted through a systematic literature review following the PRISMA protocol, based on studies retrieved from Scopus and Web of Science. The selected works were organized and synthesized using an analysis matrix, resulting in 85 primary studies. The findings indicate that XAI is mainly applied to fraud detection, credit assessment, financial auditing, and decision-support processes, with a predominance of techniques such as SHAP and LIME. Although these tools enhance transparency, limitations related to computational cost, data quality, explanation stability, and regulatory adaptation persist, highlighting the need to strengthen their integration into auditing processes.

Systematic review registration

https://osf.io/pb5cy/.

## 1 Introduction

In recent decades, digital technology has driven a sustained transformation in the financial sector, characterized by the expansion of tools such as online banking, mobile banking, digital wallets, e-commerce platforms, and cryptocurrencies. These innovations aim to optimize the speed, security, and accessibility of transactions for users, companies, and institutions. The consolidation of digital ecosystems and the progressive replacement of traditional banking methods with technological solutions reflect the magnitude of this digitalization process (Turgut et al., 2025).

In this context, artificial intelligence (AI) has assumed a central role in financial. In the accounting domain, AI has attracted increasing interest due to its potential to automate routine tasks, analyze large volumes of data, improve outcome prediction, and add strategic value to financial management (Al Lawati et al., 2024; Leocádio et al., 2024; Mishra et al., 2024; Desai, 2025b). However, many of these models, particularly those based on deep learning, operate as “black-box” systems, which hinders the understanding of their decisions and raises concerns regarding transparency, accountability, and institutional control.

These limitations are especially critical in scenarios where automated decisions have a direct impact on clients, organizations, and regulatory systems. In particular, the financial sector faces annual losses amounting to billions of dollars due to fraud, which requires systems capable not only of identifying suspicious transactions with high accuracy but also of justifying their outcomes in an understandable manner. Nevertheless, many machine learning–based models lack interpretability, which may lead to unjustified account blocks, user dissatisfaction, and risks of regulatory non-compliance (Abisha et al., 2025).

In response to this situation, Explainable Artificial Intelligence (XAI) has emerged as a solution to the opacity of intelligent systems by promoting transparent and interpretable models for various stakeholders, including data scientists, auditors, risk managers, executives, and regulatory authorities. XAI seeks to facilitate the understanding of the internal functioning of algorithms in order to ensure their alignment with ethical and regulatory frameworks (Ullah et al., 2022; Vuković et al., 2025). Its relevance has increased in highly regulated sectors, where automated decisions must be justifiable and auditable, such as in anti-money laundering systems or bias-free credit assessment processes (Desai, 2025a).

Within the financial domain, XAI has become an essential component for addressing challenges related to data privacy, regulatory harmonization, institutional responsibility, and algorithmic governance (Vuković et al., 2025). In accounting and financial auditing, in particular, XAI enables the generation of explanations that can be technically validated, interpreted by analysts, and supervised by regulatory bodies, thereby strengthening transparency, accountability, and model robustness (Baisholan et al., 2025a). In this regard, Dubey et al. (2025) emphasize that the transparency provided by XAI is fundamental for both internal auditing and external regulatory scrutiny, especially in fraud detection contexts.

From a methodological perspective, XAI integrates various approaches aimed at making complex machine learning models more understandable. Among the most widely used are feature attribution methods, grounded in game theory; local surrogate models; visualization techniques; and counterfactual explanations, which analyze how small changes in input data can alter system decisions (Ikermane et al., 2025; Mollik and Majeed, 2025).

Despite the growing interest in applying XAI in the financial sector, most of the literature has focused on domains such as healthcare and justice, whereas its systematic integration into accounting and financial auditing still exhibits conceptual and methodological gaps (Mishra et al., 2024). Consequently, there remains a need for rigorous analyses of advances, trends, prevailing approaches, and challenges associated with the implementation of XAI techniques in this field.

### 1.1 Background

The concept of explainability in artificial intelligence can be traced back to expert systems developed in the 1970s, which incorporated explicit rules to facilitate the understanding of their inference processes. However, the term “explainable artificial intelligence” (XAI) was formalized in the mid-2000s in response to the growing use of complex and opaque models (Schwalbe and Finzel, 2024). Currently, XAI constitutes a well-established research field that seeks to make AI outcomes more transparent, interpretable, and reliable through global and local explanations, thereby strengthening accountability and user trust, especially in high-risk financial sectors (Oukhouya et al., 2025).

In the specific context of auditing, Zhong and Goel (2024) analyzed the application of XAI techniques in a fraud detection case using classification models. Through the use of LIME, SHAP, and counterfactual explanations, they demonstrated how these tools reveal the reasoning behind predictions, enhancing auditors’ trust and aligning with emerging regulations on responsible AI. The authors also emphasized the role of XAI in the development of auditable, reliable, and regulation-compliant applications.

Complementarily, Cil and Yildiz (2025a) conducted a systematic review of XAI applications in the financial sector, identifying SHAP and LIME as the most frequently used methods in areas such as credit risk assessment, fraud detection, and portfolio management. Their findings indicate that the integration of XAI improves model interpretability and validation while maintaining performance levels comparable to black-box systems, with accuracy rates ranging from 88 to 99.5%, and predominantly using real-world financial data.

In specialized contexts, Alsobeh et al. (2025) applied XAI in tax auditing processes by integrating SHAP values and attention heatmaps, enabling early detection of potentially fraudulent tax returns and the generation of justifiable explanations for each alert. This approach promotes fairness, accountability, and legal compliance, supporting auditors in making well-grounded decisions. Similarly, Khan et al. (2025) analyzed model-agnostic XAI methods in finance, identifying SHAP, LIME, counterfactual explanations, and PDPs as prominent techniques, while highlighting challenges related to balancing interpretability, predictive accuracy, and computational complexity.

The development and adoption of XAI have also been strongly shaped by regulatory frameworks. Regulations such as the General Data Protection Regulation (GDPR) in the European Union and U.S. credit regulations require clear explanations of automated decisions, thereby encouraging the incorporation of explainability mechanisms in financial systems. Currently, financial institutions face interjurisdictional challenges when attempting to harmonize diverse regulatory standards, which complicates the uniform implementation of XAI (Desai, 2025b). This issue is particularly critical in areas such as credit scoring, fraud detection, and anti-money laundering, where automated decisions have direct legal implications and require high levels of interpretability (Vallarino, 2025).

Overall, the literature shows sustained progress in the development and application of XAI in the financial sector and auditing processes. Nevertheless, conceptual, technical, and regulatory challenges remain, particularly regarding method standardization, the integration of hybrid approaches, and applicable procedures. These gaps justify the need for systematic studies that synthesize existing evidence and guide future research in accounting and financial auditing.

The present study aims to systematically analyze the scientific literature on the application of Explainable Artificial Intelligence (XAI) in accounting and financial auditing, with the purpose of identifying its main areas of application, the methods employed, and the challenges associated with its implementation, thereby contributing to the theoretical and methodological consolidation of the field. To achieve this objective, the study seeks to address the following research questions:

*RQ1:* In which areas of accounting and financial auditing has Explainable Artificial Intelligence been applied?

*RQ2:* What methods are used in the implementation of XAI in accounting and financial auditing?

*RQ3:* What challenges or limitations exist in relation to the implementation of XAI in accounting and financial auditing?

## 2 Theoretical framework

**Figure 1:**
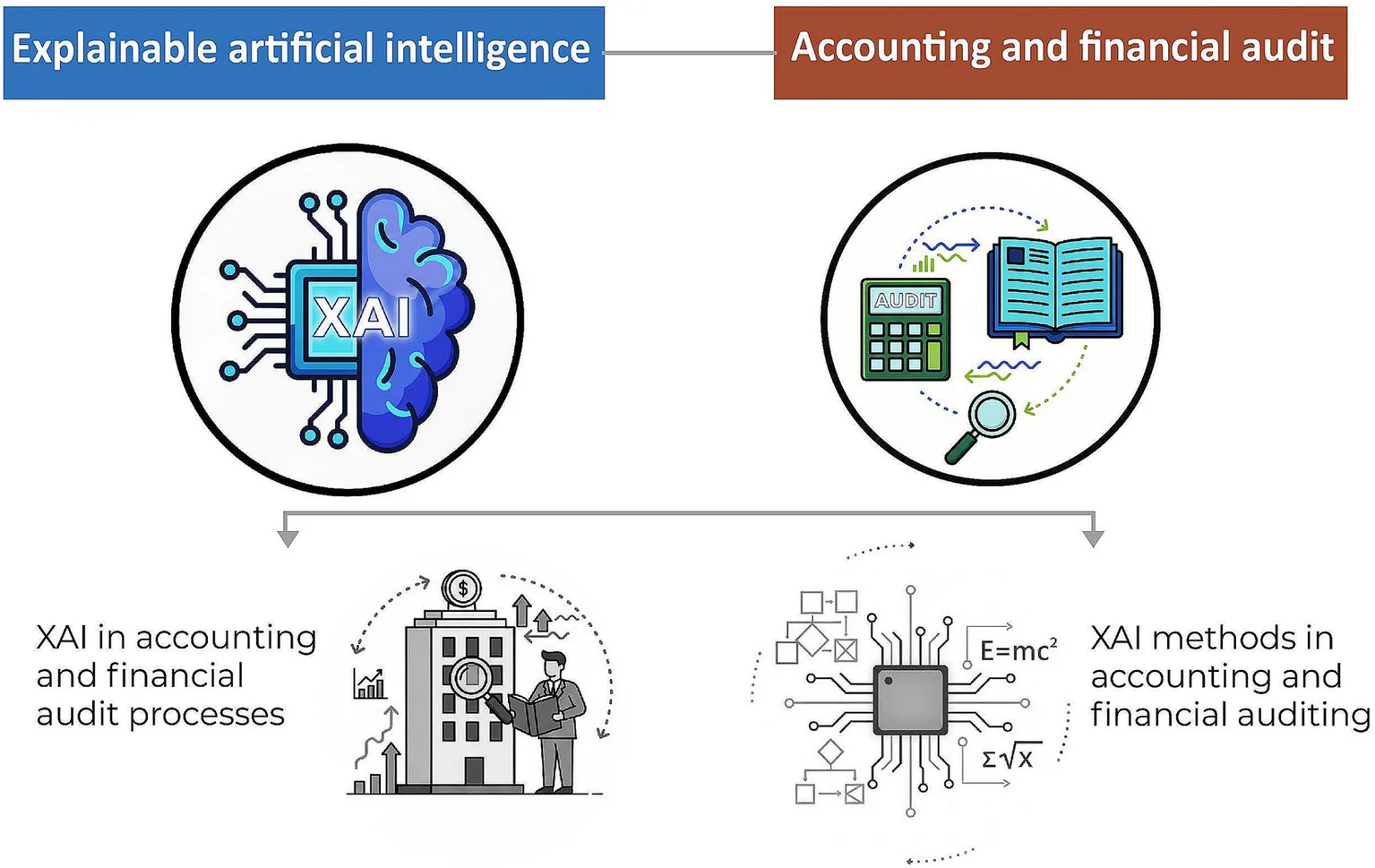
Main variables of the study.

For Figure 1 presents a conceptual diagram that synthesizes the main variables of the study: explainable artificial intelligence and accounting and financial auditing. This representation illustrates their interrelationship within auditing processes, highlighting the areas in which XAI is applied and the methods used to promote transparency, interpretability, and decision-making. Based on this framework, the study seeks to identify both the specific processes in which XAI is integrated and the explanatory techniques employed in the accounting and financial context.

Explainability and interpretability constitute central dimensions in the contemporary analysis of artificial intelligence–based systems. Explainability refers to a model’s ability to reveal its internal mechanisms by identifying the variables, relationships, and processes that influence its predictions, whereas interpretability refers to the extent to which such explanations can be understood by non-specialized human actors, such as auditors, regulators, and organizational decision-makers. In this sense, Explainable Artificial Intelligence (XAI) emerges as a field aimed at developing methods that connect algorithmic reasoning with human understanding processes, thereby reducing the gap between computational complexity and informed decision-making (Mamba et al., 2025).

From an epistemological perspective, XAI can be understood as a translation mechanism between the implicit knowledge embedded in mathematical models and the explicit knowledge required by institutional control systems. Within this framework, various techniques function as cognitive mediation devices that enable the reconstruction of the meaning of automated decisions (Mamba et al., 2025). This function is particularly relevant in normative disciplines such as auditing, where the legitimacy of knowledge depends on its verifiability and traceability.

The theoretical systematization of XAI has been addressed through taxonomies aimed at classifying explanatory mechanisms. Barredo et al. (2020) distinguish between inherently interpretable models and post-hoc approaches, and further differentiate techniques specific to deep learning based on layers, internal representations, and attention mechanisms. This classification allows explainability to be understood not as a homogeneous property, but as a theoretical continuum that encompasses different levels of transparency, ranging from fully interpretable models to highly opaque systems that require external explanatory mediation.

From a theoretical-methodological perspective, the categories proposed by Černevičienė and Kabasinskas (2024)—feature relevance, simplification, local explainability, visualization, transparent models, and integrated frameworks—reflect different modes of constructing explanatory knowledge. Each category represents a particular way of representing causality, influence, and uncertainty in financial systems, configuring an epistemological pluralism within the field of XAI.

In the domain of accounting and financial auditing, professional knowledge has historically been built upon principles such as obtaining sufficient and appropriate evidence, systematic risk assessment, the exercise of professional judgment, and responsibility toward third parties (Messier et al., 2017). These principles shape an epistemological rationality oriented toward the intersubjective validation of financial knowledge. The incorporation of artificial intelligence systems introduces a new form of technical rationality, based on statistical patterns and machine learning, which challenges the traditional foundations of assurance and verification.

In this context, XAI acts as a mechanism for articulating algorithmic rationality with the auditor’s professional rationality. By enabling complex models, such as neural autoencoders used in anomaly detection, to generate comprehensible explanations, XAI facilitates the reintegration of human judgment in automated environments. In this way, AI-based decisions can be interpreted, justified, and documented in accordance with auditing practice standards (Müller et al., 2022).

## 3 Materials and methods

The present study adopts a qualitative, documentary, and descriptive–analytical approach based on a Systematic Literature Review (SLR), aimed at rigorously identifying, analyzing, and synthesizing existing scientific evidence on the application of Explainable Artificial Intelligence in accounting and financial auditing. This methodological design makes it possible to integrate dispersed findings, evaluate trends, compare theoretical and methodological approaches, and establish a structured overview of the state of the art in the field. To ensure transparency, reproducibility, and validity, the study followed the guidelines established by the PRISMA methodology (Preferred Reporting Items for Systematic Reviews and Meta-Analyses) (Page et al., 2021), which guided the phases of identification, screening, eligibility, and inclusion of studies.

The records, databases, search criteria, filtering processes, analytical procedures, and intermediate results of the review are available on the Open Science Framework (OSF) platform and in the Supplementary material, in order to facilitate verification, replication, and future extension of the present study.

### 3.1 Sources of information and eligibility criteria

The sources of information consisted of the scientific databases Scopus and Web of Science, selected for their broad coverage, editorial rigor, and relevance in the applied and social sciences. The search strategy was applied on both platforms without temporal restrictions, given the emerging nature of research on Explainable Artificial Intelligence in accounting and financial auditing.

Studies published in any language were considered to ensure comprehensive coverage of scientific production. In the case of Web of Science, records from emerging journals were excluded, prioritizing consolidated and higher-impact sources. As eligibility criteria, scientific articles, conference papers, book chapters, and review studies were included, provided that they explicitly addressed the topic. These criteria contributed to the relevance, quality, and methodological diversity of the selected studies.

### 3.2 Search strategy

The search strategy was designed using a block-based approach, aimed at integrating terms related to explainable artificial intelligence and accounting and financial auditing. In an initial stage, the search was conducted in the Scopus database, and the preliminary results were examined in RStudio (version 4.4.2) using the Biblioshiny application to identify additional keywords, synonyms, and conceptual variants not previously considered. Based on this exploratory analysis, the search string was refined and optimized and subsequently applied to the Web of Science database.

The final search was conducted on December 15, 2025. In both platforms, the retrieved records were filtered according to the established eligibility criteria and restricted to the title, abstract, and keywords fields. This procedure strengthened thematic coherence, ensured the timeliness of the information, and guaranteed the academic quality of the selected studies (see Table 1).

**Table 1:** Search strings and retrieved results.

Database	Search string	Total
Scopus	(TITLE-ABS-KEY (“financial audit*” OR “internal audit*” OR “accounting audit*” OR “auditability” OR “financial reporting” OR “fraud detection”) AND TITLE-ABS-KEY (“XAI” OR “Explainable Artificial Intelligence” OR “Explainable AI”)) AND (LIMIT-TO (DOCTYPE, “cp”) OR LIMIT-TO (DOCTYPE, “ar”) OR LIMIT-TO (DOCTYPE, “ch”) OR LIMIT-TO (DOCTYPE, “re”))	233
Web of science	(TS = (“financial audit*” OR “internal audit*” OR “accounting audit*” OR “auditability” OR “financial reporting” OR “fraud detection”)) AND TS = (“XAI” OR “Explainable Artificial Intelligence” OR “Explainable AI”)	84
Total		317

### 3.3 Study selection process

**Figure 2:**
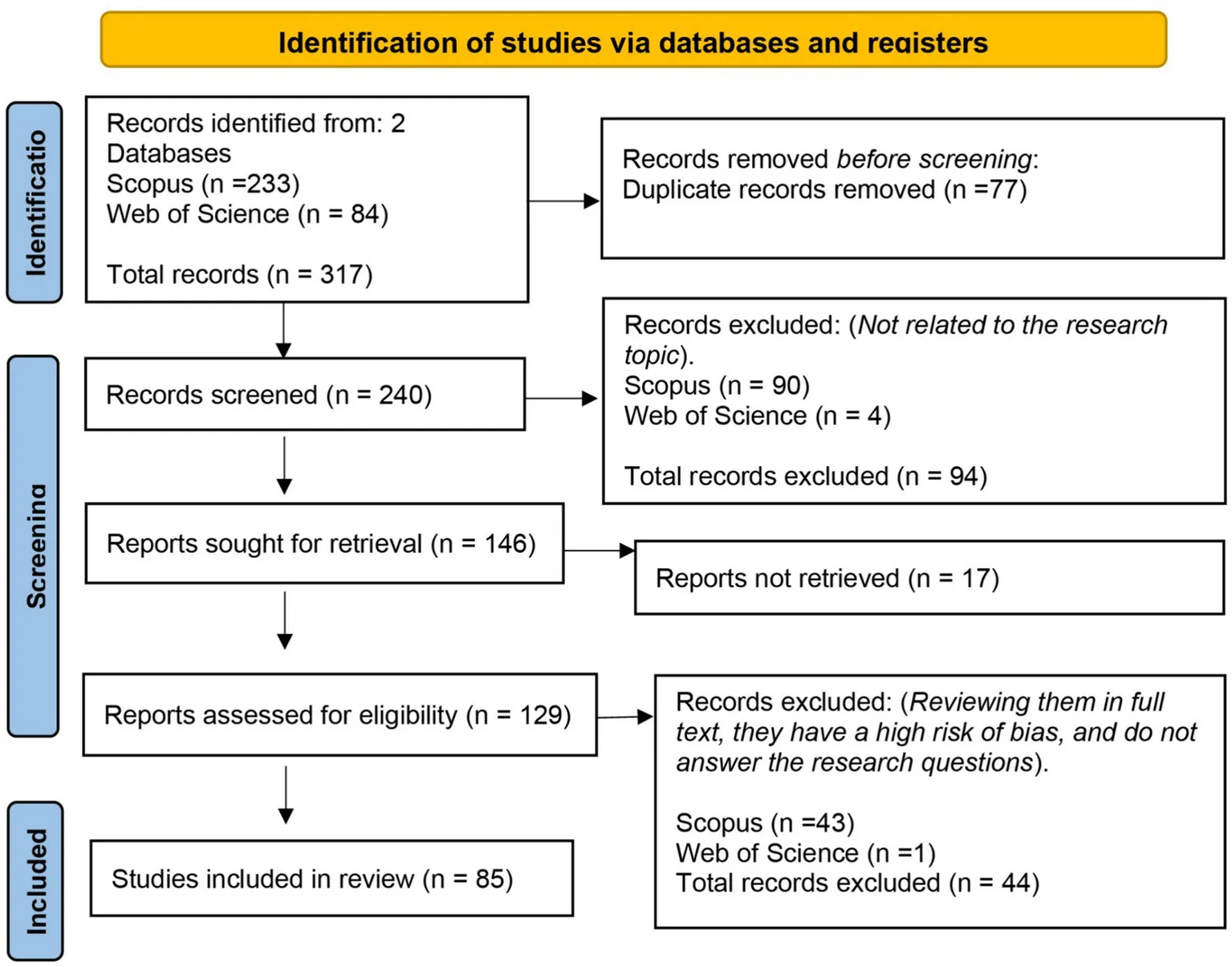
PRISMA flowchart.

The study selection process was conducted in accordance with the phases of identification, screening, eligibility assessment, and inclusion, following the PRISMA guidelines (see Figure 2). During the identification stage, 317 records were retrieved from two databases: Scopus (*n* = 233) and Web of Science (*n* = 84). Subsequently, 77 duplicate records were removed, resulting in a total of 240 studies for the initial screening based on title and abstract review.

At this stage, 94 records were excluded for not being related to the research topic, of which 90 corresponded to Scopus and 4 to Web of Science, leaving 146 studies for retrieval. Of these, 17 could not be obtained in full-text form, and therefore 129 reports were assessed at the eligibility stage. Full-text review led to the exclusion of 44 studies (43 from Scopus and 1 from Web of Science) due to a high risk of bias or their limited contribution to addressing the research questions. As a result, 85 studies met all the established criteria and were included in the systematic review.

### 3.4 Bias risk assessment

The risk of bias assessment was conducted through a consensus-based review process involving all authors. The risk of bias was minimized through a structured, multi-stage procedure. First, only databases of recognized prestige and high academic standards were selected, and emerging journals were excluded, prioritizing well-established scientific publications. In addition, only studies that applied XAI techniques were selected, excluding those that did not use these methods or did not provide clear explanations. Subsequently, each study was evaluated in two phases.

In the first phase, titles, abstracts, and keywords were reviewed to determine their direct contribution to the research topic; at this stage, each record was independently assessed by two authors, and in cases of disagreement, a third author acted as an arbiter. In the second phase, corresponding to the full-text review, the same collaborative evaluation procedure was applied, and studies that did not adequately address the research questions, deviated from the focus of the study, or presented methodological limitations and deficiencies in results assessment were excluded. This procedure contributed to strengthening the coherence, transparency, and rigor of the analysis by reducing the influence of potential biases during the final selection of studies. A detailed description of the coding, evaluation, and reviewer consensus process is provided in the Supplementary material of the study.

### 3.5 Synthesis methods

Information synthesis was carried out using a qualitative thematic analysis approach, supported by a structured matrix developed in Microsoft Excel. This matrix systematized each study’s responses to the research questions, organizing information on application domains, employed methods, and identified challenges. Once the matrix was completed, the data were grouped and categorized according to emerging conceptual and thematic patterns in order to integrate, compare, and synthesize the findings coherently. This procedure enabled the consolidation of results, facilitated cross-study analysis, and ensured a consistent interpretation of the available evidence.

## 4 Results

### 4.1 Explainable artificial intelligence (XAI) in accounting and financial auditing processes

Table 2 systematizes the application domains of explainable artificial intelligence in accounting and financial auditing, linking them to studies that document its use in different contexts. In the area of fraud detection and prevention, research conducted in diverse operational environments is grouped, including electronic payment systems, digital platforms, banking infrastructure, and corporate settings. These studies describe the use of XAI in scenarios characterized by high transaction volumes and significant risks of irregularities.

**Table 2:** Application domains and uses of XAI in accounting and financial auditing.

Application domain	Specific applications
Fraud detection and prevention	Credit card transactions (Kotrachai et al., 2023; Raval et al., 2023; Awosika et al., 2024; Azad et al., 2025; Dontha et al., 2025; Gokhale and Naik, 2025; Karim et al., 2025; Kibria et al., 2025; Kilickaya, 2025; Malhotra et al., 2025; Sowmya et al., 2025; Srivastava et al., 2025).
	Digital payments and electronic wallets (Mamba et al., 2025; Mozumder et al., 2025; Shah, 2025)
	Automated teller machines (ATMs) (Vivek et al., 2024).
	Bank account fraud and identity theft (Awosika et al., 2024; Ahmad et al., 2025; Sinha and Dubey, 2025).
	Occupational and corporate fraud (Tritscher et al., 2023, 2024; Zhong and Goel, 2024).
Credit evaluation and risk management	Credit scoring, customer solvency assessment, and portfolio management (Chavakula et al., 2025; Cil and Yildiz, 2025b; Desai, 2025b; Priya et al., 2025).
	Loan approval (Mishra et al., 2024; Cil and Yildiz, 2025b; Selvam, 2025).
	Payment prediction and financial risk forecasting (Dhanawat et al., 2024; Cil and Yildiz, 2025b; Kapale et al., 2025; Sabharwal et al., 2025; Talaat et al., 2025).
Compliance and regulatory auditing	Anti–money laundering (AML) prevention (Basani, 2024; Konstantinidis and Gegov, 2024; Sinha and Dubey, 2025; Vallarino, 2025).
	Internal and external auditing (Kiefer and Pesch, 2021; Al-Daoud and Abu-AlSondos, 2025; Aljunaid et al., 2025; Desai, 2025b).
	Cross-border monitoring (Al-Daoud and Abu-AlSondos, 2025).
	Regulatory accountability (Zhou et al., 2023; Desai, 2025b; Selvam, 2025).
Financial Auditing and financial statement analysis	Anomaly detection in financial statements (Kiefer and Pesch, 2021; Müller et al., 2022; Mishra et al., 2024; Vijayanand and Smrithy, 2025).
	Accounting error identification (Kim and Kim, 2025).
	Audit risk assessment (Mishra et al., 2024).
	Corporate fraud prediction (Zhong and Goel, 2024).
Decision-Making support and operational efficiency	Support for fraud analysts (Mill et al., 2023; Kibriya et al., 2025).
	Reduction of operational workload (Cirqueira et al., 2021).
	Prioritization of critical cases (Cirqueira et al., 2021).
	Generation of explanatory reports (Shu et al., 2024; Desai, 2025b; Srivastava et al., 2025).
	Provision of managerial insights (Mill et al., 2023).

In the domain of credit evaluation and risk management, studies focused on credit scoring, solvency analysis, loan approval, and financial risk prediction are included. Complementarily, the category of compliance and regulatory auditing encompasses research aimed at anti–money laundering prevention, internal and external audits, cross-border monitoring, and regulatory accountability, reflecting the use of XAI in supervision and regulatory control functions.

**Figure 3:**
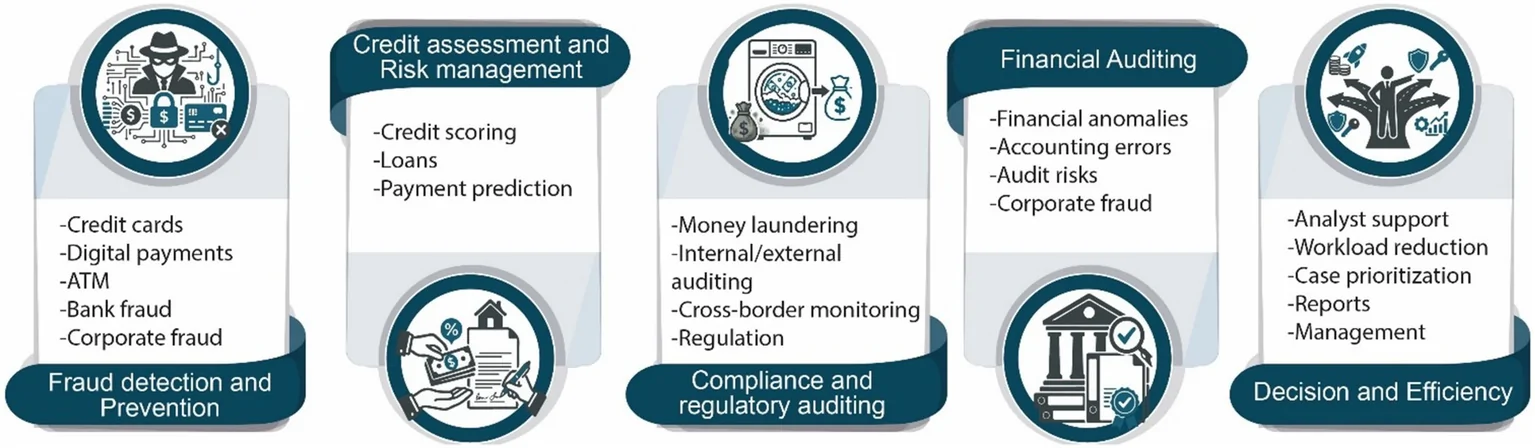
Application areas of XAI methods in accounting and auditing.

The category of financial auditing and financial statement analysis integrates studies related to anomaly detection, error identification, audit risk assessment, and corporate fraud analysis. Furthermore, the domain of decision-making support and operational efficiency includes research focused on supporting analysts, reducing operational workload, prioritizing critical cases, and generating explanatory reports (see Figure 3).

### 4.2 XAI methods used in accounting and financial auditing

Table 3 presents a classification of Explainable Artificial Intelligence (XAI) techniques identified in the analyzed literature, organized according to their functional categories and their correspondence with a theoretical taxonomy based on the framework proposed by Barredo et al. (2020). This systematization makes it possible to structure the different approaches used in the field of accounting and financial auditing, facilitating the identification of methodological patterns.

**Table 3:** Categories and XAI methods in accounting and financial auditing.

XAI categories	Methods	Taxonomy
Interpretable models	Interpretable Generalized Additive Neural Networks (IGANN), Explainable Boosting Machines (EBM), Interpretable Linear Models.	Transparent models
Local simplification	Local Interpretable Model-Agnostic Explanations (LIME), LIME Variants, Anchors, SOAR (Surrogate Optimization for Actionable Rules), Rule Extraction, Model Distillation.	Post-hoc → Simplification
Feature importance	SHapley Additive exPlanations (SHAP), Kernel SHAP, Tree SHAP, Deep SHAP, Time SHAP, Loss SHAP, Adaptive SHAP (A-SHAP), RESHAPE (Representation SHAP Explanation), SHAP-XAI, Permutation Feature Importance (PFI), Partial Dependence Variance (PDV).	Post-hoc → Feature relevance
Visualization	Partial Dependence Plots (PDP), Violin Plots, Heatmaps.	Post-hoc → Visualization
Counterfactual/causal	Diverse Counterfactual Explanations (DiCE), Structural Causal Models (SCM), Causal Graph Neural Networks (Causal GNN), CausalNex, Counterfactual Reasoning.	Post-hoc → Examples
Deep learning	Layer-wise Relevance Propagation (LRP), Integrated Gradients, Gradient × Input, Attention Mechanisms, Time SHAP.	DL-specific XAI
Reasoning	Belief–Desire–Intention (BDI), Case-Based Reasoning (CBR), SFIX (Structured Feature Interaction Explanations).	Symbolic XAI
Interactive	Human-in-the-loop	Interactivity

In the category of interpretable models, techniques such as IGANN (Modadugu et al., 2025), EBM (Gokhale and Naik, 2025; Kibriya et al., 2025), and interpretable linear models (Malhotra et al., 2025) are included. These approaches are characterized by being transparent by design, allowing a direct understanding of the relationship between input variables and model outputs. Accordingly, they fall within the taxonomy of transparent models.

The local simplification category groups methods such as LIME (Wu and Wang, 2021; Kotios et al., 2022; Nobel et al., 2024; Saini et al., 2025; Shah, 2025; Swathi et al., 2025; Tripathy et al., 2025), its variants, Anchors, SOAR, rule extraction, and model distillation. These techniques are framed within the post-hoc simplification approach, as they construct approximate and interpretable models to explain specific predictions of complex systems.

The feature importance category includes techniques such as SHAP (Bhowmik et al., 2022; Gangavarapu et al., 2024; Kasoju and Vishwakarma, 2024; Agomuo et al., 2025; Annamalai et al., 2025; Baisholan et al., 2025b; Karnavou et al., 2025), TreeSHAP, DeepSHAP (Keerthana et al., 2025), KernelSHAP (Parkar et al., 2024; Kilickaya, 2025), TimeSHAP (Bento et al., 2021), LossSHAP, A-SHAP, RESHAPE (Müller et al., 2022), SHAP-XAI (Talaat et al., 2025), PFI (Kim and Kim, 2025; Mamba et al., 2025) and PDV (Lozano-Murcia et al., 2025). These tools correspond to the post-hoc feature relevance approach, whose objective is to quantify the individual contribution of each variable to model predictions.

The visualization category includes methods such as Partial Dependence Plots (PDP) (Lozano-Murcia et al., 2025), violin plots (van Veen et al., 2026), and heatmaps (Alsobeh et al., 2025), which enable graphical representation of model behavior and variable influence within the post-hoc visualization framework.

The group of counterfactual and causal explanations comprises techniques such as DiCE (Raufi et al., 2024; Baisholan et al., 2025a), Structural Causal Models (SCM), Causal Graph Neural Networks (Causal GNN), counterfactual reasoning (Vallarino, 2025), and CausalNex (Parkar et al., 2024). These methodologies are classified as post-hoc example-based approaches, as they explain predictions through alternative scenarios or causal relationships.

The category of deep learning–specific methods groups techniques such as LRP (Tritscher et al., 2024; Priya et al., 2025), Integrated Gradients, Gradient × Input (Tritscher et al., 2024), attention mechanisms (Varatharajoo et al., 2024), and TimeSHAP (Bento et al., 2021). These methods are designed to interpret deep neural networks and are integrated within the DL-specific XAI taxonomy.

In the domain of symbolic reasoning, approaches such as BDI (Dubey et al., 2025), CBR (Desai, 2025a; Ge and Xu, 2025) and SFIX (Cil and Yildiz, 2025b) are included. These are classified as Symbolic XAI and are oriented toward generating explanations based on rules, cases, or logical structures. The interactive category incorporates the human-in-the-loop approach (Al-Daoud and Abu-AlSondos, 2025), which emphasizes active user participation in the explanation and validation processes.

**Figure 4:**
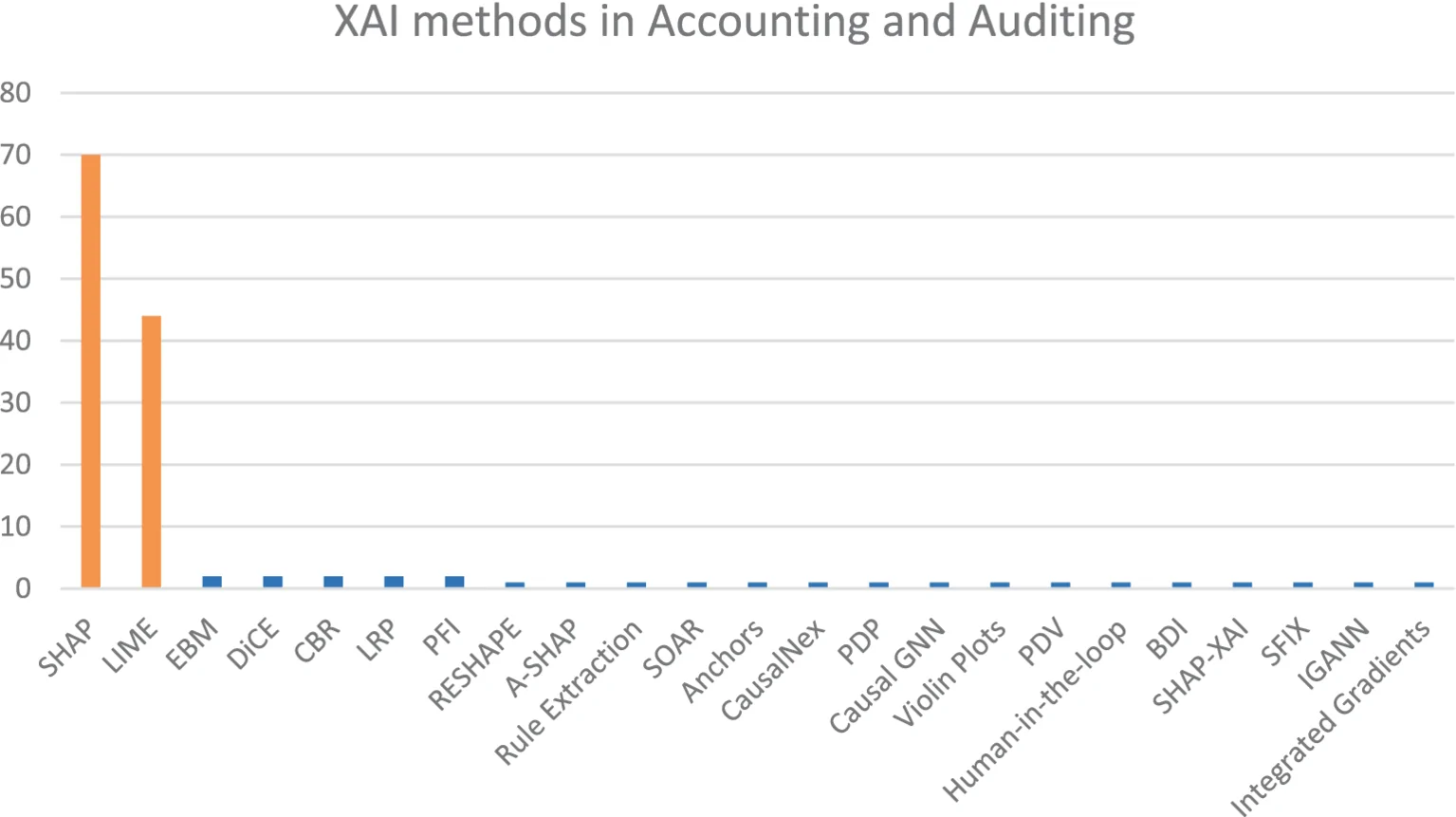
Frequency of mention of XAI methods in the literature.

In general, according to the analyzed literature, the most recurrent techniques correspond to local simplification approaches based on LIME and feature relevance approaches based on SHAP and its variants (see Figure 4). This finding suggests that these methods constitute the main references for explainability in the field of accounting and financial auditing. Their predominant frequency reflects their widespread adoption across different contexts and applications, allowing for consistent and comparable interpretations of the complex models used in financial studies.

In the reviewed studies, SHAP and LIME were identified in a total of 114 cases, representing 81.43% of all research on XAI in accounting and financial auditing. This high percentage indicates that most of the literature focuses on these methods, both because of their ability to generate local and global explanations and their ease of integration into existing auditing systems. The results suggest that, although multiple XAI techniques exist, SHAP and LIME constitute the most robust and empirically supported reference approaches.

Other methodologies, such as Anchors, SOAR, Permutation Feature Importance, Integrated Gradients, Grad-CAM, LRP, BDI, CBR, and SFIX, appear more sporadically and are mentioned as complementary approaches. These techniques provide methodological diversity and alternatives for specific contexts, but they are not explored in depth due to their lower frequency in the literature and limited evidence of practical application in accounting and auditing. Since SHAP and LIME are the most widely used methods, their mathematical models and application examples in accounting and financial auditing are presented.

### 4.3 Mathematical models of SHAP and LIME

The SHAP model was proposed in 2017 by Scott Lundberg and Su-In Lee. Its main contribution lies in the identification of a new class of additive feature attribution measures, together with the development of theoretical foundations demonstrating the existence of a unique solution within this class that satisfies a set of desirable properties (Lundberg and Lee, 2017).

SHAP values are computed based on cooperative game theory, estimating the average marginal contribution of each feature across all possible combinations of variables. Formally, the SHAP value of a feature 𝑗 is obtained by summing the difference between the model prediction with and without that feature over all possible subsets of the full set of variables, weighted by a normalization factor that accounts for the number of permutations (See Equation 1) (Ahmad et al., 2025).

This formulation enables a fair distribution of importance among features, ensuring that each variable receives a weight proportional to its actual impact on the model’s prediction. Consequently, SHAP provides a consistent and comparable measure of relevance at both local and global levels, facilitating the interpretation of complex models in financial contexts (Iqbal and Amin, 2025).

#### 4.3.1 SHAP


$$\mathrm{\phi j}(f)=\sum_{S\subseteq N\setminus \{j\}}\frac{|S|!(|N|-|S|-1)!}{|N|!}[f\;(S\cup \{j\})-f\;(S)]$$ (1)

Where:

N: total set of model features

S: subset of features that does not include j

f(S) : model prediction using only the variables in S

f(S∪{j}) : prediction after adding feature j

∣S∣ size of the subset

∣N∣ : total number of features

ϕj(f) : SHAP value of feature j

#### 4.3.2 LIME

The LIME method was proposed in 2016 by Marco Túlio Ribeiro, Sameer Singh, and Carlos Guestrin as an innovative explanation technique aimed at interpreting the predictions of any classifier in a faithful and understandable manner (Ribeiro et al., 2016). LIME locally approximates the behavior of a complex model by constructing an interpretable surrogate model, selected through the minimization of a loss function that measures the discrepancy between the two models in the neighborhood of the analyzed instance. To achieve this, it generates perturbed samples around the point of interest and evaluates the corresponding predictions, fitting a simple model that approximately represents the local decision boundary (See Equation 2). This formulation makes it possible to identify the most influential features in individual predictions, facilitating the understanding of the classification process and supporting the validation of automated decisions in financial contexts (Karim et al., 2025).


$$\hat{f}(x)=\begin{array}{cc} {\text{arg min }E}_{x^{\prime }}[L(f,g,x^{\prime })] & g\in G \end{array}$$ (2)

Where:

f(x) : original complex (black-box) model

g(x) : approximate interpretable model

G: set of interpretable models (linear regression, small decision trees, etc.)

x : analyzed original instance

$x^{\prime }$ perturbed samples around x

$L(f,g,x^{\prime })$ : loss function between f and g

$\hat{f}(x)$ : best local approximation of the original model

### 4.4 SHAP and LIME in accounting and financial auditing

In the context of accounting and financial auditing, SHAP (SHapley Additive exPlanations) stands out as one of the most widely used explainability techniques for quantifying and visualizing the contribution of each feature to the predictions of complex models. Its foundation in game theory makes it possible to decompose a model’s output into individual contributions attributable to each variable, thereby facilitating the interpretation of decisions related to fraud detection, accounting anomalies, and atypical transactions (Iqbal et al., 2025; van Veen et al., 2026).

In fraud detection systems, SHAP values enable the assessment of both the magnitude and direction of the impact of each financial variable on the classification of a transaction or entity as fraudulent or legitimate. This information is commonly represented through summary plots and violin plots, which illustrate how high or low values of specific features positively or negatively influence the estimated probability of fraud. These visual mechanisms help auditors understand the underlying reasons behind model predictions, strengthening transparency and traceability in analytical processes (Fukas et al., 2022; van Veen et al., 2026).

From a methodological perspective, Thanathamathee et al. (2024) highlight the use of SHAP in XGBoost-based models, in which SHAP values are computed for each feature and each instance in the dataset. Based on these values, instance importance is obtained by summing the absolute SHAP values, allowing the identification of the most influential samples in the learning process. This SHAP-based weighting approach is particularly useful in imbalanced datasets, which are typical in fraud scenarios, as it guides training toward more informative cases and improves model accuracy.

In the field of accounting anomaly detection, Müller et al. (2022) propose the RESHAPE model, which employs Shapley values to transform the opaque outputs of autoencoder neural networks into explanations that are understandable to auditors. This model disaggregates the contribution of each accounting attribute in the identification of irregularities, presenting information at an aggregated level. As a result, auditors can understand not only the existence of an anomaly but also the specific variables that generated it, which is essential for supporting professional judgment, meeting documentation requirements, and strengthening review processes.

Additionally, Iqbal et al. (2025) note that SHAP enables both global and individual explanations, facilitating the identification of latent patterns associated with fraud, even in environments where features are anonymized. This capability is particularly relevant in financial systems where data protection limits direct access to sensitive information, while institutions remain obligated to justify their decisions to users and regulatory bodies.

From a regulatory and institutional perspective, Al-Daoud and Abu-AlSondos (2025) emphasize that SHAP is used to provide local explanations for black-box models, supporting internal audit preparation and external regulatory accountability. In contexts such as customer disputes, cross-border monitoring, and supervision within financial institutions of the Gulf Cooperation Council (GCC), algorithmic transparency constitutes a regulatory mandate. The authors also highlight the use of SHAP to assess the stability of explanations across model updates and within human-in-the-loop frameworks, ensuring trust and auditability.

Similarly, LIME (Local Interpretable Model-Agnostic Explanations) represents one of the most widely used techniques for generating local explanations of complex models in accounting and financial auditing. Its approach is based on constructing interpretable models around individual predictions by generating perturbations in input features and fitting a simple model that locally approximates the behavior of the “black-box” system. This procedure makes it possible to identify the contribution of each financial variable in specific decisions related to fraud detection and anomaly identification (Gonzalez, 2024; Keerthana et al., 2025).

LIME operates by slightly perturbing the values of transaction features, obtaining the corresponding predictions from the main model, and weighting these observations according to their proximity to the analyzed case. Through this process, an interpretable model is constructed that reveals how each feature influences the probability that a transaction is classified as fraudulent or legitimate. This approach enables analysts to understand why a specific instance was flagged as irregular and supports the verification or correction of potential misclassifications (Gonzalez, 2024; Keerthana et al., 2025).

In empirical applications, Vihurskyi (2024) employs LIME in credit card fraud detection using a European Kaggle dataset. In this study, data are preprocessed through duplicate removal and class balancing using the SMOTE technique to correct imbalances between fraudulent and legitimate transactions. Subsequently, models such as Decision Trees and Random Forests are trained, and LIME generates localized explanations during the prediction phase. These explanations detail the most influential features in each classification, contributing to improved interpretability, trust, and transparency among stakeholders.

In the domain of unsupervised anomaly detection, Kiefer and Pesch (2021) propose an indirect approach that combines LIME with supervised models. In this framework, an XGBoost model is trained to globally approximate the behavior of unsupervised anomaly detection algorithms, and LIME is then applied to generate local, model-agnostic explanations. These explanations are post-processed to provide auditors and data scientists with a selective understanding of the features responsible for each anomaly, enhancing interpretability in environments where original models lack direct explanatory mechanisms.

Complementarily, Damanik and Liu (2025) apply LIME in stacked fraud detection systems, where multiple base classifiers, such as Random Forests and Decision Trees, feed a meta-learner responsible for the final prediction. In this context, LIME enables the analysis of how each base model interprets data and how their outputs are integrated into the global decision. This approach facilitates the identification of key attributes driving predictions, providing actionable information for financial institutions and improving decision-making transparency.

From an institutional perspective, the application of LIME in auditing strengthens accountability by enabling auditors and regulators to understand the financial factors underlying automated classifications. By generating understandable local explanations, LIME supports result validation, bias detection, and continuous model improvement, which are essential in highly regulated environments (Gonzalez, 2024; Keerthana et al., 2025).

**Figure 5:**
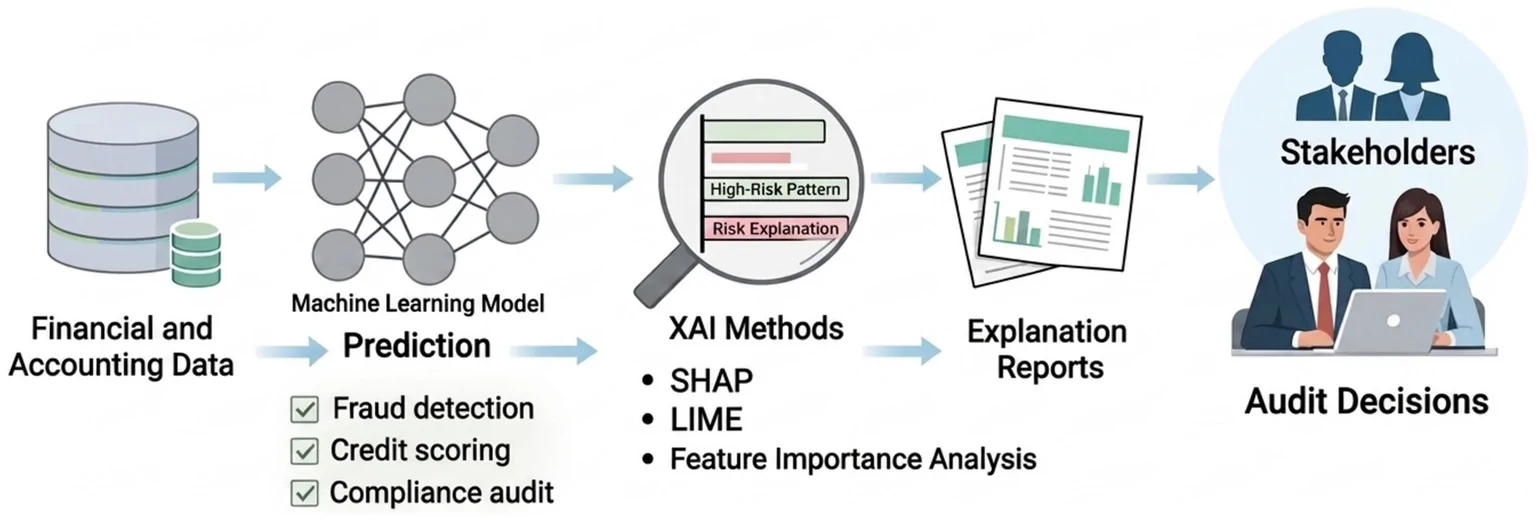
Explanation of XAI processes in accounting and financial auditing.

In the broader context of accounting and financial auditing, the incorporation of explainable artificial intelligence techniques is embedded within a structured process that begins with the collection and preparation of financial data, continues with the training of machine learning models, and culminates in the generation of automated predictions or alerts (see Figure 5). These techniques operate in a post-modeling phase, acting as a bridge between computational outputs and human interpretation. Through this approach, system outputs cease to be merely numerical values or opaque classifications and are transformed into understandable explanations that make it possible to identify the variables and patterns underlying each decision.

The application of SHAP and LIME within this workflow enables auditors to access detailed information about model behavior at different levels of analysis. These tools process the generated predictions and produce interpretable representations that facilitate technical validation, documentary support, and the communication of results to supervisory bodies and stakeholders. In this way, explanations become a central component of the system, strengthening traceability, internal control, and coherence between automated findings and professional judgment.

*C*ompared with traditional approaches based on manual reviews, limited sampling, and static rules, XAI-supported systems offer a substantial advantage by combining high processing capacity with analytical transparency. Without explanatory mechanisms, artificial intelligence models operate as “black boxes,” making it difficult to justify decisions, increasing regulatory risks, and limiting institutional trust. In contrast, the integration of XAI into the auditing process reduces exclusive reliance on human analysis, expands review coverage, improves early detection of irregularities, and ensures that automated results can be understood, evaluated, and substantiated in accordance with the technical and ethical principles of accounting and financial auditing.

### 4.5 Challenges and limitations of XAI in accounting and financial auditing

One of the main challenges in the application of XAI in accounting and financial auditing is its predominant reliance on correlational rather than causal relationships (Parkar et al., 2024; van Veen et al., 2026). Data anonymization and the absence of reference causal graphs limit semantic interpretation and result validation (Baisholan et al., 2025a, 2025b; van Veen et al., 2026), hindering a deeper understanding of the mechanisms underlying automated predictions.

From a methodological perspective, the scarcity of independent real-world datasets restricts the assessment of model generalization and increases the risk of overfitting (Aljunaid et al., 2025; Ge and Xu, 2025; Vadlamudi et al., 2025). Many studies prioritize predictive accuracy, relegating systematic analyses of interpretability and thereby limiting understanding of decision-making processes (Iqbal et al., 2025).

Related to its practical implementation, explainable AI techniques particularly post-hoc methods such as SHAP and LIME, entail high processing costs, scalability issues, and sensitivity to small data perturbations. The calculation of individual contributions of each variable for every instance in the dataset can be computationally expensive, particularly in large datasets or those with numerous features. This creates scalability issues, making implementation difficult in high-frequency or real-time environments. Additionally, sensitivity to small perturbations in the data can generate variations in the explanations, affecting the stability and reliability of the results (Al-Daoud and Abu-AlSondos, 2025; Aljunaid et al., 2025; Chavakula et al., 2025; Keerthana et al., 2025). These constraints affect their applicability in real-time or high-frequency auditing environments, where latency is critical (Kibriya et al., 2025; Kilickaya, 2025).

Data quality and structure represent another significant limitation. Financial datasets are often imbalanced, noisy, and characterized by missing values (Mill et al., 2023; Aljunaid et al., 2025; Chavakula et al., 2025; Iqbal and Amin, 2025). Although balancing and anonymization techniques are necessary, they may distort original distributions and weaken variable interpretability (Baisholan et al., 2025b). Moreover, these conditions affect the fidelity of the explanations generated by XAI models, as the estimated importance or contribution values may not accurately reflect the true influence of each variable on the predictions, reducing the reliability of the explanations for decision-making in auditing and accounting.

From an organizational perspective, the lack of auditor-oriented explanatory interfaces and the shortage of specialized personnel hinder effective XAI adoption (Baisholan et al., 2025a; Desai, 2025b; Karnavou et al., 2025). This is compounded by digital literacy gaps, which limit many professionals’ ability to critically interpret generated explanations (Zhong and Goel, 2024). These limitations affect auditors’ confidence in the results provided by XAI models, reducing the effectiveness of decision-making. They also highlight the need for human-AI collaboration mechanisms, where auditors can interact with explanations, validate hypotheses, and adjust criteria according to their professional judgment, strengthening the integration of AI into organizational processes and ensuring that the interpretation of results is reliable and applicable in practical contexts.

At the regulatory and ethical level, challenges persist related to regulatory heterogeneity, privacy protection, the absence of consolidated standards, and risks of algorithmic bias (Raval et al., 2023; Aljunaid et al., 2025; Vallarino, 2025). These tensions generate institutional uncertainty regarding the scope and validity of automated explanations (Fukas et al., 2022).

The dynamic nature of financial fraud introduces concept drift phenomena that reduce model stability and require frequent retraining (Al-Daoud and Abu-AlSondos, 2025). The scarcity of labeled data, the vulnerability to adversarial attacks that could manipulate generated explanations, and the lack of standardized criteria or robust frameworks to evaluate the quality of explainability further limit the cumulative development of the field (Lozano-Murcia et al., 2025). These limitations make it difficult to ensure the fidelity, consistency, and reliability of explanations, highlighting the need to implement metrics and evaluation frameworks that allow validation of both the stability and robustness of explanations in auditing and financial accounting environments.

## 5 Discussion

Explainable artificial intelligence has gained increasing importance as algorithmic systems have been incorporated into different professional and organizational fields. Its use has moved beyond technological contexts and has expanded into areas where decisions require transparency, justification, and accountability, such as accounting and financial auditing. Abdo-Salloum and Chehade (2026) indicate that artificial intelligence is transforming accounting and auditing practices through task automation, improved financial analysis, irregularity detection, and decision-making support. From this perspective, explainability becomes especially relevant because it enables AI-generated results to be understood, interpreted, and used by accountants, auditors, and financial managers, even when they do not possess advanced technical knowledge of programming, technology, or specialized software. In this way, XAI enables these professionals to assess the factors that influence a prediction, compare the results with their professional judgment, support their decisions before third parties, and strengthen trust in financial information.

Unlike other regulated environments, accounting and auditing require decisions to be understandable, verifiable, and defensible before clients, regulators, investors, and other stakeholders. Cumming et al. (2023) emphasize that financial and accounting research should move toward future debates related to information quality, trust, governance, and the evolution of financial systems. Therefore, XAI represents a technical tool for interpreting models and, at the same time, a practical resource for improving audit quality, accountability, institutional transparency, and financial governance.

The results show that the application of Explainable Artificial Intelligence in accounting and financial auditing is mainly concentrated in fraud detection, credit risk assessment, regulatory compliance, financial statement analysis, and decision-support processes. In the area of fraud, the literature emphasizes the use of XAI in credit card transactions, digital payments, identity theft, and corporate fraud (Kotrachai et al., 2023; Tritscher et al., 2023, 2024; Awosika et al., 2024; Zhong and Goel, 2024), reflecting its operational relevance for financial institutions. Complementarily, studies on credit scoring, loan approval, and risk prediction indicate growing interest in integrating explainable models into strategic financial evaluation processes (Cil and Yildiz, 2025b; Priya et al., 2025).

Nallakaruppan et al. (2024) highlight that explainable models can help financial institutions and regulators better understand the factors that influence credit risk predictions, thereby strengthening trust, fairness, and data-driven decision-making. In the regulatory domain, XAI applications in anti-money laundering, internal auditing, and accountability mechanisms are highlighted as tools supporting institutional supervision and transparency (Kiefer and Pesch, 2021; Basani, 2024; Al-Daoud and Abu-AlSondos, 2025).

Regarding the methods employed, the analyzed literature reveals a clear predominance of post-hoc techniques, particularly SHAP and LIME, oriented toward local explanations and feature relevance identification. However, although SHAP and LIME maintain a dominant presence in the reviewed literature, recent studies show that complementary approaches are already being developed to overcome the limitations of explanations based solely on feature attribution. Cil et al. (2026) propose an XAI approach for audit opinion classification using feed-forward neural networks, in which the SFIX framework enables an intrinsic and multidimensional assessment of financial features, distinguishing it from post-hoc methods such as SHAP and LIME. Li et al. (2026) propose a financial fraud detection system based on causal-temporal asynchrony, where the model explanation is linked to patterns consistent with the fraud triangle and with a continuous auditing logic.

Faccia (2026) addresses XAI in auditing as a bridge between predictive fraud models and regulatory standards, emphasizing the need for explanations that are reviewable, documentable, and aligned with accountability. Bhatti (2026), in a review on AI ethics in finance, emphasizes that explainability is part of a broader issue involving governance, transparency, trust, and human oversight. Thivaios et al. (2026) also recognize that modern financial fraud detection is moving toward more diverse architectures, such as graph-based models, multimodal approaches, federated learning, and interpretable AI. These advances are complemented by recent proposals that use large language models to transform complex chains of risk evidence into understandable narratives compatible with regulatory standards, as in the case of GraphCredit. Therefore, the predominance of SHAP and LIME should be understood as a relevant but still transitional stage within a broader agenda that includes causal explainability, inherently interpretable models, graph-based reasoning, and LLM-supported narrative explanations.

From an operational perspective, the reviewed studies also highlight limitations associated with XAI adoption in real-world environments. Desai (2025b) warns that divergent regulations, the need for human interpretation, and computational challenges hinder large-scale implementation, particularly in resource-constrained institutions. These constraints are related to the high computational cost of methods in complex models (Iqbal and Amin, 2025)

Furthermore, data quality emerges as a critical factor that can affect both the fidelity of explanations and the reliability of decisions derived from XAI models. Imbalanced, incomplete, or noisy financial data can lead to misinterpretations of the models, impacting auditors’ confidence and the consistency of findings, as noted in previous studies (Mill et al., 2023; Aljunaid et al., 2025). Additionally, biases present in the data may be inadvertently reinforced by XAI techniques, generating significant ethical risks, such as unfair or discriminatory decisions in risk management and auditing (Baisholan et al., 2025b; Zhong and Goel, 2024).

### 5.1 Practical implications

These aspects show that XAI methods can provide concrete practical benefits in accounting and financial auditing, especially by enabling auditors, accountants, and internal control officers to understand the variables that influence a fraud alert, a risk estimate, a credit classification, or an automated recommendation. From this perspective, XAI can be understood as a bridge between algorithmic outputs and professional judgment, as it facilitates the comparison of model predictions with available evidence, the interpretation of the factors that influence a decision, and the support of professional conclusions before third parties.

In professional practice, these tools can support evidence documentation, the review of unusual transactions, the prioritization of risk areas, and the justification of decisions before clients, audit committees, regulators, and investors. For auditors, this involves using explanations as support to strengthen critical evaluation, rather than as a substitute for professional judgment. For financial institutions, it involves establishing protocols for data validation, bias review, model performance monitoring, and human oversight. For regulators, explainability can serve as a criterion for requiring traceability, auditability, and accountability in the use of algorithmic systems applied to sensitive financial decisions. However, its adoption requires verifying data integrity and quality, assessing whether the generated explanations are understandable to non-technical users, and establishing controls over privacy, transparency, and professional responsibility. Therefore, the sustainable implementation of XAI depends not only on model performance, but also on internal validation protocols, user training, human oversight, and alignment with the regulatory and ethical frameworks applicable to the financial-accounting environment.

### 5.2 Limitations of the study

One of the limitations of this study arises from the high methodological diversity among the reviewed studies, including differences in research designs, data sources, analytical techniques, and evaluation criteria. This heterogeneity limited the possibility of conducting direct comparisons and hindered the establishment of uniform standards for assessing the effectiveness and reliability of XAI approaches in accounting and financial auditing. In addition, variations in sample sizes, validation procedures, and technical implementations may affect the consistency of the reported findings.

### 5.3 Future research directions

Future research could focus on addressing the current limitations of XAI in accounting and financial auditing, promoting greater methodological standardization and transparency. Given that the available studies are still limited, it is essential to develop shared benchmarks, unified reporting guidelines, and publicly accessible datasets, which would facilitate more robust comparisons across studies and allow for validation of different techniques.

Advanced explainability approaches should be explored, including causal explainability, graph-based methods, counterfactual reasoning, and logic-based linear models (LLM), as well as other emerging interpretable models, evaluating their applications in accounting and auditing contexts. Such research could implement hybrid evaluation frameworks that combine technical performance metrics, fidelity, and stability with usability and user-centered trust assessments.

Additionally, longitudinal and field studies are needed to examine the long-term effects of XAI adoption on audit quality, professional judgment, and organizational governance. Comparative analyses across regulatory environments and institutional contexts would help understand how to integrate XAI effectively and responsibly. These initiatives would not only allow comparisons between existing methods such as SHAP and LIME and emerging techniques, but also provide a solid foundation to develop standardized evaluation metrics, assess practical applicability, and extend the implementation of XAI in real-world auditing and accounting environments.

## 6 Conclusion

The analyzed evidence indicates that the application of Explainable Artificial Intelligence in accounting and financial auditing is mainly concentrated in areas related to fraud detection and prevention, credit risk assessment, regulatory compliance, financial statement analysis, and decision-support processes. These contexts correspond to critical functions for the reliability of financial information and institutional stability. The centrality of these domains in the reviewed literature suggests that XAI has been primarily incorporated in settings where transparency, decision traceability, and accountability are essential to ensure the credibility of automated systems.

From a methodological perspective, the results reveal a predominance of post-hoc approaches, especially techniques such as SHAP and LIME, aimed at explaining complex models through feature contribution analysis and local explanations. These tools stand out for their adaptability to different algorithms and their ability to produce interpretations accessible to auditors, regulators, and end users. Although inherently interpretable models, causal methods, and deep learning–specific techniques are also identified, their use is less frequent. This pattern reflects a tendency to prioritize predictive performance while complementing it with explanatory mechanisms, rather than adopting fully transparent architectures from the outset.

In terms of implementation, the literature reveals the persistence of technical, organizational, and regulatory challenges that condition the effective use of XAI in auditing. Key limitations include the high computational cost of certain techniques, the instability of some explanations, dependence on data quality and availability, and difficulties in translating technical outputs into operational information for professionals. These challenges are compounded by regulatory requirements, jurisdictional differences, and resource constraints in some institutions. Overall, these factors indicate that the consolidation of XAI in auditing requires not only technological advances but also the strengthening of institutional, educational, and governance capacities.

## Data Availability

The original contributions presented in the study are included in the article/Supplementary material, further inquiries can be directed to the corresponding author.
